# Defining and characterizing sustained remission in patients with rheumatoid arthritis

**DOI:** 10.1007/s10067-017-3923-z

**Published:** 2017-12-09

**Authors:** Jeffrey R. Curtis, Mona Trivedi, Boulos Haraoui, Paul Emery, Grace S. Park, David H. Collier, Girish A. Aras, James Chung

**Affiliations:** 1 University of Alabama at Birmingham, 510 20th Street South, Birmingham, AL 35294 USA; 2 Amgen Inc., Thousand Oaks, CA USA; 3 Centre Hospitalier de l’Université de Montréal, Montreal, Canada; 4 Leeds Institute of Rheumatic and Musculoskeletal Medicine, University of Leeds, Chapel Allerton Hospital, NIHR Leeds Biomedical Research Centre, Leeds Teaching Hospitals NHS Trust, Leeds, UK

**Keywords:** Etanercept, Methotrexate, Remission, Rheumatoid arthritis

## Abstract

The objective of this study is to characterize stability and clinical features of patients with rheumatoid arthritis (RA) in sustained remission. Combination therapy with methotrexate and tumor necrosis factor inhibitors (TNFi) has increased remission rates in RA but optimal regimens to maintain remission are unknown. We describe Study of Etanercept And Methotrexate in Combination or as Monotherapy in Subjects with Rheumatoid Arthritis (SEAM-RA) and data from a run-in period of longitudinal observation. Patients in Simplified Disease Activity Index (SDAI) remission (score ≤ 3.3) receiving etanercept and methotrexate were screened and had to maintain remission over 3 run-in visits/24 weeks before randomization to combination therapy or withdrawal of etanercept or methotrexate. Baseline characteristics were examined for predictive factors for maintaining remission. As of November 2016, 141 patients have enrolled; of these, 64 have been randomized, 34 were ineligible after run-in, and 43 are in run-in period; 70% have completed run-in. Enrolled and randomized patients, respectively, had mean (standard deviation [SD]) disease duration 11.0 (8.6) and 12.6 (9.7) years; mean (SD) duration of etanercept use 4.2 (3.8) and 4.9 (4.2) years; mean (SD) methotrexate dose 15.9 (4.8) and 15.5 (4.9) mg/week; and mean (SD) SDAI scores 1.5 (0.9) and 1.4 (0.8). At enrollment, 73% and 63% were in Boolean remission based on 28 joints and 66/68 joints, respectively. No enrollment characteristic predicted successful completion of run-in. Two-thirds of patients considered to be in remission at enrollment sustained remission through 24 weeks. Baseline characteristics of enrolled patients and those who completed run-in were comparable.

**Electronic supplementary material:**

The online version of this article (10.1007/s10067-017-3923-z) contains supplementary material, which is available to authorized users.

## Introduction

The American College of Rheumatology (ACR) [1] and the European League Against Rheumatism (EULAR) [2] both recommend low disease activity (LDA) or remission as the goal of treatment for patients with rheumatoid arthritis (RA). ACR and EULAR define remission based on Simplified Disease Activity Index (SDAI) score ≤ 3.3 or Boolean remission (tender joint count [TJC] ≤ 1, swollen joint count [SJC] ≤ 1, C-reactive protein [CRP] ≤ 1 mg/dL, and patient global assessment [PGA] ≤ 1 on a 0–10 scale) [3]. The US Food and Drug Administration (FDA) supports remission as an endpoint in clinical trials and recommends following patients in remission to provide information on the durability of the remission response [4]. The benefits of maintaining good disease control have been amply demonstrated, and there is evidence that remission may lead to better outcomes compared to LDA [5–8]. The introduction of biologic therapies, including tumor necrosis factor inhibitor (TNFi) medications, for the treatment of moderate to severe RA has made remission more achievable than was previously seen with conventional synthetic disease-modifying antirheumatic drugs (csDMARDs), e.g., methotrexate. Currently, there is no clinical definition for maintenance of remission in routine clinical practice, which is an important issue with a disease with inherent fluctuations.

Many aspects of RA remission need to be explored. What is the difference between physician perceptions of remission vs the reality of remission based on composite scores? Does the historic use of Disease Activity Score based on 28 joints (DAS-28) as a clinical measure skew perceptions compared to the use of more restrictive criteria with SDAI remission? How stable is SDAI remission? Does it last weeks, months, or even years?

With more patients achieving remission using combination therapy with a csDMARD such as methotrexate plus a TNFi, patients and clinicians increasingly question the need for continued use of both agents to maintain good disease control. Key issues in the decision to discontinue treatment include maintenance of efficacy, long-term safety and tolerability, and costs [9, 10]. ACR/EULAR guidelines for the treatment of patients with RA suggest strategies to manage a patient who is in remission: (1) taper the corticosteroids; (2) taper the csDMARD; (3) taper the TNFi medication, non-TNFi biologic DMARD (bDMARD), or tofacitinib; and (4) do not discontinue all RA therapies [1, 2]. These recommendations are based on low to moderate levels of evidence, and there is clearly a need for well-designed studies to evaluate DMARD tapering or discontinuation in patients in remission. Overall, studies that examined discontinuing a TNFi medication in patients with LDA or in remission showed that discontinuation sometimes leads to worsening of disease and an increased number of flares [9–15]. The extent of worsening appears to depend on the stage of disease (early vs established disease) and the depth of disease control (LDA vs remission).

The aim of the Study of Etanercept And Methotrexate in Combination or as Monotherapy in Subjects with Rheumatoid Arthritis (SEAM-RA; study 20110186; ClinicalTrials.gov #NCT02373813) is to provide information of practical utility to the physician who is considering simplifying or discontinuing the treatment regimen in patients with RA on the combination of a TNFi medication and methotrexate after sustained good disease control. SEAM-RA has been designed to assess the clinical impact of discontinuing methotrexate or etanercept in patients receiving combination therapy with these medications who have achieved remission and may help to identify markers that predict the maintenance of response after medication withdrawal. The clinical hypothesis being tested is that etanercept monotherapy is superior to methotrexate monotherapy for maintaining remission as defined by SDAI in patients with RA who were on etanercept plus methotrexate therapy. The ability of etanercept monotherapy and combination therapy to maintain remission will be described, although no formal statistical analysis will be conducted between these groups.

Three key features of the study are the definition of remission, the duration of remission before any therapy is discontinued, and the comparison of etanercept and methotrexate as monotherapy in the study design. SDAI assessments are used to identify patients in remission; an SDAI score ≤ 3.3 is considered to be the most stringent definition of remission and is being used for SEAM-RA [16]. Patients must maintain SDAI-defined remission on combination therapy with etanercept plus methotrexate for 24 weeks during the open-label run-in period before randomization to etanercept or methotrexate monotherapy. Patients are randomized to one of three treatment arms: etanercept plus methotrexate combination therapy, etanercept monotherapy, or methotrexate monotherapy. The study design provides an opportunity to characterize in detail RA patients who are in remission based on SDAI. This report will provide information about early enrollees in SEAM-RA, including how often SDAI remission was maintained over 24 weeks, how many patients moved in and out of remission, and for those who failed to maintain remission, and how close they were to re-achieving remission. These data will inform both the SEAM-RA trial and future withdrawal studies and their inclusion criteria.

## Patients and methods

### Study design

**Fig. 1:**
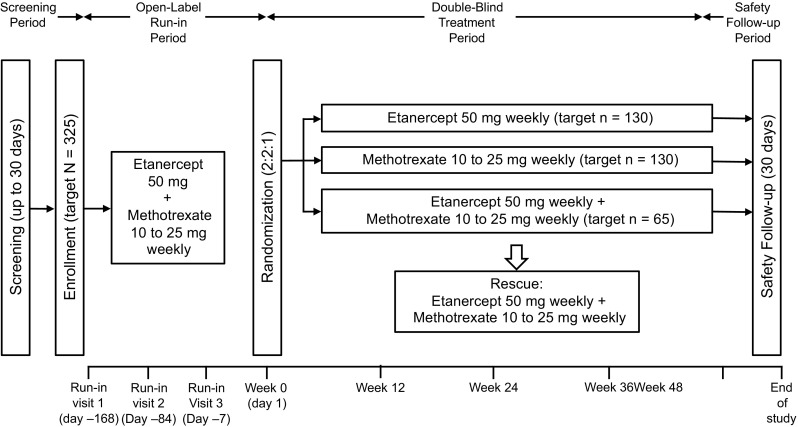
Study schema

SEAM-RA is a phase 3, multicenter, randomized, double-blind, controlled, withdrawal study. The study comprises a 30-day screening period, a 24-week open-label run-in period, a 48-week double-blind treatment period, and a 30-day safety follow-up period (Fig. 1). During the run-in period, patients receive open-label etanercept and methotrexate at the same dose they were receiving during screening. At the end of the run-in period, eligible patients are randomly assigned (2:2:1) to etanercept 50 mg weekly by subcutaneous (SC) injection plus oral placebo (planned *n* = 130), oral methotrexate at 10 to 25 mg weekly plus SC placebo (planned n = 130), or etanercept 50 mg weekly SC plus oral methotrexate 10 to 25 mg weekly (planned *n* = 65).

Data for this study are not publicly available because the study is ongoing and remains blinded.

### Eligibility criteria

Patients are evaluated for eligibility at screening (for participation in the run-in period) and at the end of the run-in period (for participation in the double-blind period). Key inclusion criteria at screening include the following: age ≥ 18 years, history of RA consistent with ACR and EULAR classification criteria, history of moderate to severe RA in the opinion of the investigator, very good disease control for ≥ 6 months in the opinion of the investigator, SDAI score ≤ 3.3 at screening (at the beginning of the run-in period), receiving etanercept at 50 mg weekly for RA for ≥ 6 months prior to the first run-in visit, and receiving a stable dose of methotrexate at 10 to 25 mg weekly for ≥ 6 months.

To be eligible for entry into the double-blind period, patients must have had an SDAI score ≤ 3.3 just prior to randomization at run-in visit 3 and cannot have the following: any clinically significant change in eligibility criteria during the run-in period, SDAI score > 3.3 and ≤ 11 on 2 or more visits during the run-in period, or SDAI score > 11 at any time during the run-in period. This definition would, for example, allow patients to have an SDAI score between 3.3 and 11 at run-in visit 2, with the recognition that some patients might have a short-term flare that was not persistent or have clinical parameters with an SDAI that was close to but did not meet the remission definition (i.e., a “near-miss”).

### Study endpoints

The primary endpoint of the SEAM-RA trial is SDAI remission (score ≤ 3.3) at week 48 without disease worsening before week 48. Disease worsening is defined as SDAI score > 3.3 and ≤ 11 on 2 consecutive visits at least 2 weeks apart; or SDAI > 3.3 and ≤ 11 on 3 or more separate visits; or SDAI > 11 at any time. Secondary efficacy endpoints include SDAI, DAS-28 with erythrocyte sedimentation rate (DAS-28-ESR), DAS-28-CRP, and Clinical Disease Activity Index (CDAI) scores and changes from baseline at all study time points; SDAI remission at all time points; Boolean remission at all time points; percentage of patients with worsening disease based on SDAI scores; time to disease worsening; and time to recapture remission after rescue therapy is given. Definitions of Boolean remission include 28 joints, 28 joints plus feet and ankles (as recommended by ACR/EULAR for assessments of remission [3]), and 66/68 joints. An additional endpoint is the proportion of patients who are eligible to continue in the study after run-in. Safety endpoints include incidence of all adverse events, serious adverse events, and laboratory parameters.

## Results

### Patients

#### Screened patients

Over half of all subjects were ineligible to participate in the study at screening (Supplemental Fig. S1). The most common reasons for screen failure were elevated SDAI score, positive hepatitis B or hepatitis C testing, and laboratory abnormalities.

#### Enrolled patients

As of November 1, 2016, a total of 141 patients have completed screening and enrolled in the study. The patient population was predominantly female (72%) and white (85%) (Table 1). Notably, mean SDAI, CDAI, and DAS-28 scores were consistent with patients in remission. Among the 141 enrolled patients, 73% 66% and 63% had achieved remission based on the Boolean definition with 28 joints, 28 joints plus feet and ankle joints, and 66/68 joints, respectively (Table 1). No additional patients would have failed screening because of lack of sustained remission if the criteria for remission permitted SDAI scores > 3.3 or Boolean remission.

**Table 1:** Demographic and clinical characteristics at enrollment

Characteristic	All enrolled patients (*N* = 141)	Patients who failed run-in (*N* = 34)	Currently randomized patients (*N* = 64)
Age, mean (SD) years	57.3 (10.4)	58.1 (11.8)	57.6 (10.2)
Sex, *n* female (%)	102 (72.3)	23 (67.6)	44 (68.8)
Race, *n* (%)			
White	120 (85.1)	28 (82.4)	54 (84.4)
Black/African American	15 (10.6)	5 (14.7)	7 (10.9)
Other	6 (4.3)	1 (2.9)	3 (4.7)
Duration of RA, mean (SD) years	11.0 (8.6)	11.4 (8.7)	12.6 (9.7)
Methotrexate dose, mean (SD) mg/week	15.9 (4.8)	15.6 (4.3)	15.5 (4.9)
Duration of etanercept use, mean (SD) years	4.2 (3.8)	4.1 (3.6)	4.9 (4.2)
SDAI, mean (SD) score	1.5 (0.9)	1.6 (0.9)	1.4 (0.8)
CDAI, mean (SD) score	1.2 (0.9)	1.3 (0.9)	1.1 (0.8)
DAS-28-ESR, mean (SD) score	2.0 (0.7)	2.1 (0.7)	1.9 (0.7)
DAS-28-CRP, mean (SD) score	1.2 (0.2)	1.2 (0.3)	1.2 (0.2)
Boolean remission (28 joints), *n* (%)	103 (73.0)	24 (70.6)	46 (71.9)
Boolean remission (28 joints plus feet/ankles) *n* (%)	93 (66.0)	22 (64.7)	42 (65.6)
Boolean remission (66/68 joints), *n* (%)	89 (63.1)	20 (58.8)	40 (62.5)

*CDAI* Clinical Disease Activity Index, *CRP* C-reactive protein, *DAS*-*28* Disease Activity Score based on 28 joints, *ESR* erythrocyte sedimentation rate, *RA* rheumatoid arthritis, *SD* standard deviation, *SDAI* Simplified Disease Activity Index

#### Patients who entered the run-in period

**Fig. 2:**
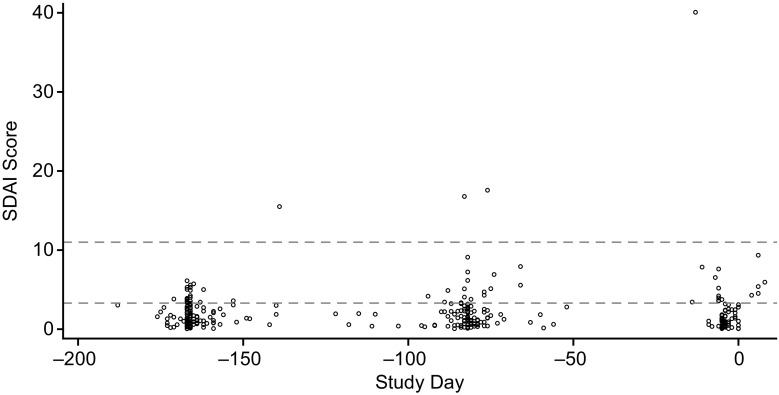
SDAI scores at 3 run-in visits. Individual patient SDAI scores during the 3 run-in visits are shown at approximately − 168 days (24 weeks prior to planned randomization on day 1), − 84 days (12 weeks prior to randomization), and day − 7 (just prior to randomization). Dashed lines indicate SDAI scores of 3.3 and 11. SDAI = Simplified Disease Activity Index

**Fig. 3:**
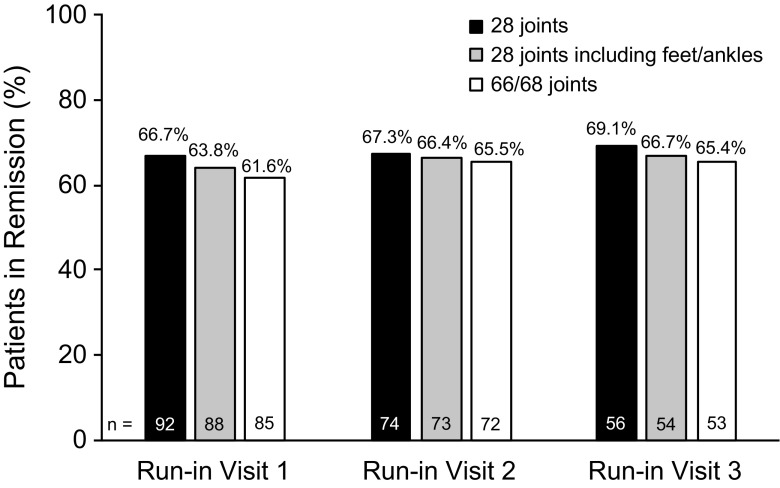
Boolean remission at run-in visits. The percentages of patients with remission using Boolean definitions based on 28 joints (black bars), 28 joints and including feet and ankle joints (gray bars), and 66/68 joints (white bars) are shown. *n* = number of patients in Boolean remission

**Fig. 4:**
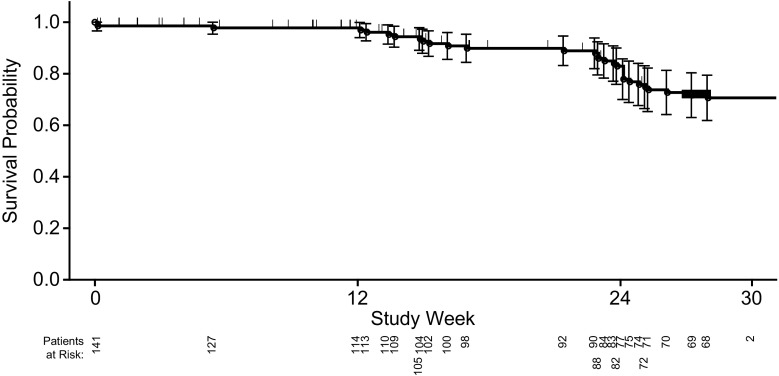
Proportion of patients remaining in the study through the run-in period. Tick marks represent censored observations, and error bars represent 95% confidence intervals

Of 141 enrolled patients, 43 are in the run-in period. The mean (standard deviation [SD]) SDAI score was 1.9 (1.5) at run-in visit 1, 2.3 (2.7) at run-in visit 2, and 2.4 (4.4) at run-in visit 3. SDAI scores and all definitions of Boolean remission were highly correlated across all 3 run-in visits (*P* < 0.001 for all correlations), and patients in remission based on Boolean definitions had lower SDAI scores (Table 2). The rate of remission was relatively stable across the run-in visits. Most patients remained in SDAI remission (score ≤ 3.3) at each run-in visit, but some were recategorized as LDA (score between 3.4 and 11.0), and a few had more severe flares (Fig. 2). Rates of remission based on Boolean definitions were lower compared with SDAI (Table 1) and were also relatively stable across the run-in visits, with the definition based on 28 joints consistently higher across definitions (Fig. 3). Approximately 30% of patients either discontinued from the study or failed to maintain SDAI remission through the 3 run-in visits, with most run-in failures occurring around run-in visit 3 (Fig. 4). Of the 27 patients who failed to meet SDAI remission criteria at any time prior to run-in visit 3 and had their final disposition known, 14 (52%) failed SDAI remission criteria and were not eligible for randomization. The remaining 13 (48%) subsequently were able to regain SDAI remission and met all other inclusion criteria at run-in visit 3 and were eligible for randomization to the main trial.

**Table 2:** Spearman correlation analysis for SDAI scores and Boolean definitions of remission

Boolean remission definition	SDAI		
	*n*	*r*	*P* value
Boolean (28 joints)			
Run-in visit 1	138	− 0.690	< 0.001
Run-in visit 2	110	− 0.731	< 0.001
Run-in visit 3	81	− 0.736	< 0.001
Boolean (28 joints plus feet/ankles)			
Run-in visit 1	138	− 0.630	< 0.001
Run-in visit 2	110	− 0.720	< 0.001
Run-in visit 3	81	− 0.696	< 0.001
Boolean (66/68 joints)			
Run-in visit 1	138	− 0.660	< 0.001
Run-in visit 2	110	− 0.737	< 0.001
Run-in visit 3	81	− 0.705	< 0.001

*r* Spearman correlation coefficient, *SDAI* Simplified Disease Activity Index

Univariate logistic regression analyses were performed to identify potential predictors of completing the run-in period. Covariates at the time of enrollment included sex, ethnicity, race, age, body mass index, weight, height, tobacco use, methotrexate dose, duration of RA, duration of methotrexate use, duration of etanercept use, SDAI score, CDAI score, DAS-28-ESR, DAS-28-CRP, all definitions of Boolean remission, TJC and SJC (28 joints, 66/68 joints), physician global assessment, PGA, CRP, ESR, and Health Assessment Questionnaire Disability Index. Proportions or means of all the covariates at Baseline did not reveal any significant differences between those who failed the run-in phase and those who were randomized.

#### Patients who completed run-in and were randomized

A total of 64 patients have been randomized to methotrexate monotherapy, etanercept monotherapy, or etanercept plus methotrexate combination therapy in the double-blind treatment period.

## Discussion

### Interim study results

Although SEAM-RA is still enrolling patients, information from the first patients screened and enrolled in the study, and an examination of patient selection at different study sites are enlightening. Of 141 patients who completed screening and enrolled in the study, 64 maintained remission for the 24-week run-in period and have been randomized. Statistical analyses have so far not revealed any demographic or clinical characteristics that predict successful completion of the run-in period (i.e., maintenance of SDAI remission for 24 weeks). In general, patients who completed run-in and were randomized were not significantly different from the subset of patients who failed to complete the run-in period.

Over half of all screened subjects failed to meet eligibility criteria. The high number of screen failures due to SDAI score may be a reflection of physicians being unfamiliar with SDAI criteria and the depth of SDAI remission compared to DAS-28 remission. No single component of the SDAI score consistently contributed to elevations in SDAI score at the time of screen failure. The overall screen failure rates for study sites did not seem to improve over time, and screen failures due to increases in SDAI score were higher in sites active > 12 months compared with those that were active ≤ 12 months, suggesting that sites were exhausting the best candidates for the trial (i.e., those with the deepest and most sustained remission) within the first year that the site was open, and few additional patients newly attained remission over time given the stringent criteria required for SEAM-RA. Additionally, while SDAI score ≤ 3.3 is the remission definition proposed by ACR/EULAR, it may not be widely used by rheumatologists as a part of regular disease assessments for RA patients, and this lack of familiarity may also be a contributing factor to the screen failure rate based on SDAI. For future real-world trials that might be more permissive and mirror the types of RA patients for whom a rheumatologist would reasonably consider stopping therapy and where a validated remission definition is not required for potential regulatory claims, a more permissive and liberal inclusion may be used (e.g., SDAI score < 11 with SJC ≤ 1).

Although patients are allowed to have minor disease fluctuations that result in elevation of SDAI scores above 3.3 and up to 11 during the first two run-in visits, allowing some natural variability in disease activity, this laxity is not permitted at run-in visit 3. An SDAI score above 3.3 at the third run-in visit results in termination of the patient from the study. For the patients who failed because of an elevated SDAI score at run-in visit 3, the SDAI scores ranged between 3.4 and 9.5; notably, all values were within the range of LDA (SDAI score < 11). Indeed, no patients with “near-misses” for SDAI remission who initially met remission criteria at run-in visit 1 were ultimately excluded. However, reassuringly, even for the patients who failed SDAI remission criteria before run-in visit 3, almost half (48%) regained remission and continued in the trial, supporting the utility of following these patients over time since many were again in remission at their next visit.

### Studies that informed SEAM-RA study design

The SEAM-RA study design was informed by prior studies of etanercept or methotrexate reduction or discontinuation after achieving good disease control on the combination. Studies including PRIZE [17], PRESERVE [18], and DOSERA [19] have characterized the role of etanercept in inducing and maintaining LDA in RA patients. Importantly, these studies demonstrated that a significantly greater proportion of patients on combination etanercept plus methotrexate therapy maintains low disease states compared to patients taking methotrexate alone. The DOSERA study demonstrated that most patients whose disease activity worsens after drug withdrawal are able to return to LDA [19]. Other studies provided insights regarding the role of etanercept as monotherapy in the maintenance of low disease states. In the CAMEO study, patients who achieved LDA (DAS-28-ESR < 3.2) at month 6 had similar disease activity at 24 months whether they discontinued methotrexate or remained on combination therapy. Combination treatment led to lower disease activity scores compared to etanercept monotherapy in patients with moderate to high disease activity at randomization (DAS-28-ESR > 3.2) [20]. In the COMET study [21], 50% of patients who discontinued methotrexate from combination therapy with methotrexate plus etanercept had achieved DAS-28 remission after 2 years, which was similar to the 57% rate of DAS-28 remission in patients who remained on combination therapy. Collectively, these studies have shown that etanercept may be required to maintain disease control and that etanercept may be sufficient as monotherapy in maintaining good disease control. Etanercept is particularly well suited to be used as monotherapy because of the lack of observed neutralizing antibodies [22]. Sustained disease remission with etanercept alone could potentially lead to a reduction in polypharmacy and undesired side effects.

### Measures of remission

Most of the studies that have examined DMARD discontinuation in patients in remission used DAS-28 to assess disease activity [9–15]. While the DAS-28 is an easy and useful tool, the weight of the indicator of inflammation (ESR or CRP) is high in the DAS-28 calculation, which may exaggerate the rate of remission in patients receiving therapies that interfere with the acute phase response [16]. Additionally, DAS-28 is not as sensitive to detect changes in disease activity in the lower ranges, i.e., LDA and remission, whereas SDAI is sensitive to changes in the lower ranges. The primary endpoint in our study is the percentage of patients who achieve remission based on SDAI criteria; other methods of assessing disease severity (DAS-28-ESR, DAS-28-CRP, CDAI), as well as remission via Boolean criteria will also be evaluated. SDAI and CDAI are considered to have the most stringent criteria for remission [16]. SEAM-RA therefore uses the most stringent clinical remission criteria, which most closely represent a true remission of disease.

An additional feature of this study is the duration of remission: only patients in good disease control for ≥ 6 months before enrollment (based on clinician judgment) and SDAI remission throughout a 24-week run-in period are randomized, for a total of at least 1 year of stable therapy and good disease control before withdrawal of any therapy. This approach was used to reflect clinical practice. Previous trials assessing withdrawal of therapy have used short periods of treatment duration before withdrawal of either etanercept or methotrexate. Given that the duration of time in remission has been shown in some studies to predict successful withdrawal [23, 24], this clinical trial attempts to take this into account and consolidates or withdraws therapy only after a patient has had at least 1 year of stable treatment with combination methotrexate plus TNFi and very good disease control. Maintenance of remission for the 48-week treatment period without disease flare will be assessed for the primary endpoint (SDAI remission). In contrast to previous studies in which remission was assessed at a single time point at the end of the study, this study will assess remission every 12 weeks and at every unscheduled disease assessment visit. Patients must remain in remission throughout the course of the study and at the final week 48 time point to be considered as maintaining remission in this study.

### Withdrawal of methotrexate vs etanercept

An alternative to discontinuing a bDMARD is to discontinue the csDMARD from combination therapy. Methotrexate has been a mainstay of RA therapy for decades, with established efficacy and safety profiles. However, a recent study showed that most patients with RA had predominantly negative implicit attitudes toward methotrexate [25], and patients may therefore prefer discontinuing methotrexate over a TNFi medication. This study will characterize the adverse events associated with methotrexate use that could be reduced or eliminated after methotrexate withdrawal, such as fatigue, malaise, oral ulcers, alopecia, and liver and gastrointestinal toxicities [26, 27]. Discontinuation of methotrexate may be difficult when used in combination with some TNFi medications, such as anti-TNF monoclonal antibodies, as it has been shown to reduce the incidence of anti-drug antibodies, which can compromise bDMARD efficacy [28]. The development of neutralizing anti-drug antibodies is associated with loss of efficacy, and if clinicians increase the dose to overcome the loss of efficacy, patients may face an increased risk of adverse events [29] and increased cost. Also, the addition of methotrexate to the anti-TNF monoclonal antibody infliximab delays the decline in serum concentrations of infliximab [30].

Etanercept does not require coadministration with methotrexate to reduce the incidence of anti-etanercept antibodies [22] or delay declines in etanercept serum concentration. Anti-drug antibodies that develop with the use of etanercept have no known clinical significance [22, 31], and neutralizing anti-drug antibodies have not been reported with the use of etanercept [22]. Therefore, discontinuation of methotrexate from combination therapy of etanercept plus methotrexate may not reduce the efficacy or serum concentrations of etanercept or result in the development of clinically significant anti-etanercept antibodies.

### Additional elements of SEAM-RA

Across RA clinical trials, there is variability in the definitions of disease flares, the number of flares allowed, and whether patients are discontinued from the study after a flare. In SEAM-RA, patients are allowed to have flares of a specified number and severity, but are not considered to be in remission if they exceed this narrow definition. SEAM-RA only allows complete withdrawal of either etanercept or methotrexate, without considering tapering doses. SEAM-RA includeds a run-in period to ensure that patients are in stable remission prior to withdrawal of any therapy. The appropriate duration of the run-in period and the most efficient number of run-in visits have not been established, but will be informed by the results of SEAM-RA.

### Conclusions

The ability to achieve remission with new targeted therapies in many patients has been an exciting and recent development in the treatment of RA. Because rates of remission were low with early therapies, there has been little need to refine definitions and assessments of remission until recently. The optimal approach to consolidating therapy after achieving stable remission remains to be adequately characterized. The results of SEAM-RA will provide important information of practical consideration for determining therapy for maintenance of remission in clinical practice.

## Electronic supplementary material

ESM 1 (DOCX 26 kb)

## References

[1] Singh JA, Saag KG, Bridges SL, Jr., Akl EA, Bannuru RR, Sullivan MC, Vaysbrot E, McNaughton C, Osani M, Shmerling RH, Curtis JR, Furst DE, Parks D, Kavanaugh A, O’Dell J, King C, Leong A, Matteson EL, Schousboe JT, Drevlow B, Ginsberg S, Grober J, St Clair EW, Tindall E, Miller AS, McAlindon T (2016) 2015 American College of Rheumatology Guideline for the Treatment of Rheumatoid Arthritis. Arthritis Rheumatol 68:1–26, 1, DOI: 10.1002/art.39480 10.1002/art.39480 26545940

[2] Smolen JS, Landewe R, Breedveld FC, Buch M, Burmester G, Dougados M, Emery P, Gaujoux-Viala C, Gossec L, Nam J, Ramiro S, Winthrop K, de Wit M, Aletaha D, Betteridge N, Bijlsma JW, Boers M, Buttgereit F, Combe B, Cutolo M, Damjanov N, Hazes JM, Kouloumas M, Kvien TK, Mariette X, Pavelka K, van Riel PL, Rubbert-Roth A, Scholte-Voshaar M, Scott DL, Sokka-Isler T, Wong JB, van der Heijde D (2014). EULAR recommendations for the management of rheumatoid arthritis with synthetic and biological disease-modifying antirheumatic drugs: 2013 update. Ann Rheum Dis.

[3] Felson DT, Smolen JS, Wells G, Zhang B, van Tuyl LH, Funovits J, Aletaha D, Allaart CF, Bathon J, Bombardieri S, Brooks P, Brown A, Matucci-Cerinic M, Choi H, Combe B, de Wit M, Dougados M, Emery P, Furst D, Gomez-Reino J, Hawker G, Keystone E, Khanna D, Kirwan J, Kvien TK, Landewe R, Listing J, Michaud K, Martin-Mola E, Montie P, Pincus T, Richards P, Siegel JN, Simon LS, Sokka T, Strand V, Tugwell P, Tyndall A, van der Heijde D, Verstappen S, White B, Wolfe F, Zink A, Boers M (2011). American College of Rheumatology/European League Against Rheumatism provisional definition of remission in rheumatoid arthritis for clinical trials. Ann Rheum Dis.

[4] US Food & Drug Administration. Guidance for industry. Rheumatoid arthritis: developing drug products for treatment http://www.fda.gov/ucm/groups/fdagov-public/@fdagov-drugs-gen/documents/document/ucm354468.pdf. Accessed June 20, 2017

[5] Curtis JR, Liu M, Rebello S, Trivedi M, Lesperance T, Li Y, Accortt N (2016). Impact of sustained remission on risk for infection in patients with rheumatoid arthritis enrolled in a US registry [abstract OP0259]. Ann Rheum Dis.

[6] Radner H, Smolen JS, Aletaha D (2014). Remission in rheumatoid arthritis: benefit over low disease activity in patient-reported outcomes and costs. Arthritis Res Ther.

[7] Aletaha D, Funovits J, Breedveld FC, Sharp J, Segurado O, Smolen JS (2009). Rheumatoid arthritis joint progression in sustained remission is determined by disease activity levels preceding the period of radiographic assessment. Arthritis Rheum.

[8] Smolen JS, Han C, van der Heijde DM, Emery P, Bathon JM, Keystone E, Maini RN, Kalden JR, Aletaha D, Baker D, Han J, Bala M, St Clair EW, Active-Controlled Study of Patients Receiving Infliximab for the Treatment of Rheumatoid Arthritis of Early Onset Study Group (2009) Radiographic changes in rheumatoid arthritis patients attaining different disease activity states with methotrexate monotherapy and infliximab plus methotrexate: the impacts of remission and tumour necrosis factor blockade. Ann Rheum Dis 68:823–827, 6, DOI: 10.1136/ard.2008.090019 10.1136/ard.2008.090019 18593759

[9] Atzeni F, Benucci M, Talotta R, Masala IF, Sarzi-Puttini P, Govoni M (2016) What are the dangers of biological therapy discontinuation or dose reduction strategies when treating rheumatoid arthritis? Expert Rev Clin Pharmacol:1–9 10.1080/17512433.2016.1234374 27634311

[10] Tanaka Y, Hirata S (2013). Is it possible to withdraw biologics from therapy in rheumatoid arthritis?. Clin Ther.

[11] Navarro-Millan I, Sattui SE, Curtis JR (2013). Systematic review of tumor necrosis factor inhibitor discontinuation studies in rheumatoid arthritis. Clin Ther.

[12] Tanaka Y, Hirata S (2014). Intensive intervention can lead to a treatment holiday from biological DMARDs in patients with rheumatoid arthritis. Drugs.

[13] Emamikia S, Arkema EV, Gyori N, Detert J, Chatzidionysiou K, Dougados M, Burmester GR, van Vollenhoven R (2016). Induction maintenance with tumour necrosis factor-inhibitor combination therapy with discontinuation versus methotrexate monotherapy in early rheumatoid arthritis: a systematic review and meta-analysis of efficacy in randomised controlled trials. RMD Open.

[14] Kuijper TM, Lamers-Karnebeek FB, Jacobs JW, Hazes JM, Luime JJ (2015). Flare rate in patients with rheumatoid arthritis in low disease activity or remission when tapering or stopping synthetic or biologic DMARD: a systematic review. J Rheumatol.

[15] Fautrel B, Den Broeder AA (2015). De-intensifying treatment in established rheumatoid arthritis (RA): why, how, when and in whom can DMARDs be tapered?. Best Pract Res Clin Rheumatol.

[16] Smolen JS, Aletaha D (2014) Scores for all seasons: SDAI and CDAI. Clin Exp Rheumatol 32:S-75-79 25365093

[17] Emery P, Hamoudeh M, FitzGerald OM, Combe B, Gaylord S, Williams T, Bukowski J, Pedersen R, Koenig AS, Vlahos B (2012). Induction of remission in patients with up to 12 months of moderate-to-severe rheumatoid arthritis symptoms treated with etanercept plus methotrexate over 52 weeks [abstract 2549]. Arthritis Rheum.

[18] Smolen JS, Nash P, Durez P, Hall S, Ilivanova E, Irazoque-Palazuelos F, Miranda P, Park MC, Pavelka K, Pedersen R, Szumski A, Hammond C, Koenig AS, Vlahos B (2013). Maintenance, reduction, or withdrawal of etanercept after treatment with etanercept and methotrexate in patients with moderate rheumatoid arthritis (PRESERVE): a randomised controlled trial. Lancet.

[19] van Vollenhoven R, Franck-Larsson K, Leirisalo-Repo M, Uhlig T, Jansson M, Larsson E, Hutchinson K, Østergaard M (2013). In rheumatoid arthritis patients with stable low disease activity on methotrexate plus etanercept, continuation of etanercept at 50 mg or 25 mg weekly are both clinically superior to discontinuation: results from a randomized, 3-arm, double-blind study [abstract FRI0185]. Ann Rheum Dis.

[20] Pope JE, Haraoui B, Thorne JC, Vieira A, Poulin-Costello M, Keystone EC (2014). The Canadian methotrexate and etanercept outcome study: a randomised trial of discontinuing versus continuing methotrexate after 6 months of etanercept and methotrexate therapy in rheumatoid arthritis. Ann Rheum Dis.

[21] Emery P, Breedveld F, van der Heijde D, Ferraccioli G, Dougados M, Robertson D, Pedersen R, Koenig AS, Freundlich B, Combination of M, Etanercept in Early Rheumatoid Arthritis Trial G (2010). Two-year clinical and radiographic results with combination etanercept-methotrexate therapy versus monotherapy in early rheumatoid arthritis: a two-year, double-blind, randomized study. Arthritis Rheum.

[22] Enbrel® (etanercept) prescribing information. Immunex Corporation, Thousand Oaks, CA. 2016

[23] Prince FH, Bykerk VP, Shadick NA, Lu B, Cui J, Frits M, Iannaccone CK, Weinblatt ME, Solomon DH (2012). Sustained rheumatoid arthritis remission is uncommon in clinical practice. Arthritis Res Ther.

[24] Navarro-Millan I, Chen L, Greenberg JD, Pappas DA, Curtis JR (2013). Predictors and persistence of new-onset clinical remission in rheumatoid arthritis patients. Semin Arthritis Rheum.

[25] Linn AJ, Vandeberg L, Wennekers AM, Vervloet M, van Dijk L, van den Bemt BJ (2016). Disentangling rheumatoid arthritis patients’ implicit and explicit attitudes toward methotrexate. Front Pharmacol.

[26] Katchamart W, Trudeau J, Phumethum V, Bombardier C (2009). Efficacy and toxicity of methotrexate (MTX) monotherapy versus MTX combination therapy with non-biological disease-modifying antirheumatic drugs in rheumatoid arthritis: a systematic review and meta-analysis. Ann Rheum Dis.

[27] Yazici Y (2010). Long-term safety of methotrexate in the treatment of rheumatoid arthritis. Clin Exp Rheumatol.

[28] Garces S, Demengeot J, Benito-Garcia E (2013). The immunogenicity of anti-TNF therapy in immune-mediated inflammatory diseases: a systematic review of the literature with a meta-analysis. Ann Rheum Dis.

[29] Murdaca G, Spano F, Contatore M, Guastalla A, Penza E, Magnani O, Puppo F (2016). Immunogenicity of infliximab and adalimumab: what is its role in hypersensitivity and modulation of therapeutic efficacy and safety?. Expert Opin Drug Saf.

[30] Klotz U, Teml A, Schwab M (2007). Clinical pharmacokinetics and use of infliximab. Clin Pharmacokinet.

[31] Hsu L, Armstrong AW (2013). Anti-drug antibodies in psoriasis: a critical evaluation of clinical significance and impact on treatment response. Expert Rev Clin Immunol.

