# Supplementary material for: Defining and characterizing sustained remission in patients with rheumatoid arthritis
Source: Clin Rheumatol. 2017 Dec 9;37(4):885–93. doi: 10.1007/s10067-017-3923-z (PMC5880849; doi:10.1007/s10067-017-3923-z)
Supplement: Supplementary file 1 — (DOCX 26 kb) [file 10067_2017_3923_MOESM1_ESM.docx]

**SUPPLEMENTAL MATERIALS**

**Defining and Characterizing Sustained Remission in Patients With Rheumatoid Arthritis**

Jeffrey R. Curtis, Mona Trivedi, Boulos Haraoui, Paul Emery, Grace S. Park,
David H. Collier, Girish A. Aras, James Chung

**Supplemental Figure S1.** Patient flow.
